# *Bacillus licheniformis*-Fermented Products Reduce Diarrhea Incidence and Alter the Fecal Microbiota Community in Weaning Piglets

**DOI:** 10.3390/ani9121145

**Published:** 2019-12-13

**Authors:** De-Yu Hung, Yeong-Hsiang Cheng, Wei-Jung Chen, Kuo-Feng Hua, Arkadiusz Pietruszka, Andrzej Dybus, Chuan-Shun Lin, Yu-Hsiang Yu

**Affiliations:** 1 Department of Biotechnology and Animal Science, National Ilan University, Yilan 26047, Taiwan; deyu0976579758@gmail.com (D.-Y.H.); yhcheng@ems.niu.edu.tw (Y.-H.C.); wjchen@niu.edu.tw (W.-J.C.); kuofenghua@gmail.com (K.-F.H.); 2 Department of Monogastric Animal Sciences, West Pomeranian University of Technology, 70-310 Szczecin, Poland; arkadiusz.pietruszka@zut.edu.pl; 3 Department of Genetics, West Pomeranian University of Technology, 70-310 Szczecin, Poland; andrzej.dybus@zut.edu.pl; 4 Animal Technology Laboratories, Agricultural Technology Research Institute, Miaoli 350-53, Taiwan; csl@mail.atri.org.tw

**Keywords:** *Bacillus licheniformis*, diarrhea, piglet, fermented product, microbiota

## Abstract

**Simple Summary:**

Antibiotics have been commonly used worldwide as growth promoters and for prophylactic treatment of diarrhea in weaning piglets. However, The European Union has banned the use of antibiotic growth promoters in animal production. Therefore, finding alternative solutions for preventing diarrhea in weaning piglets is urgent. Modulation of gut microbiota composition by probiotics has a beneficial effect on animal health. In this study, we assessed the effects of *Bacillus licheniformis*-fermented products on diarrhea incidence and the fecal microbiome composition in weaning piglets. Results showed that *B. licheniformis*-fermented products could improve diarrhea incidence and the fecal microbiota community in weaning piglets. These findings indicate that *B. licheniformis*-fermented products have the potential for development as feed additives and use as possible substitutes for antibiotics to prevent postweaning diarrhea in the pig industry.

**Abstract:**

Prophylactic use of antibiotics in-feed has been effective in decreasing the incidence of diarrhea in weaning piglets. However, the overuse of antibiotics as prophylactic or therapeutic agents in animal feed leads to the evolution of drug-resistant bacteria and antibiotic residues in pigs. This study investigated the effects of *Bacillus licheniformis*-fermented products on diarrhea incidence and the fecal microbial community in weaning piglets. A total of 120 crossbred piglets with an average initial body weight of 9.87 ± 1.43 kg were randomly allotted to four dietary treatments consisting of three replicate stalls with 10 piglets in each. The dietary treatments comprised a basal diet as control, control plus 1 g/kg or 4.5 g/kg of *B. licheniformis*-fermented products, and control plus 30 mg/kg antibiotics (bacitracin methylene disalicylate). Results showed that 4.5 g/kg of *B. licheniformis*-fermented product supplementation significantly reduced diarrhea incidence in weaning piglets. Principal coordinate analysis and a heatmap of species abundance indicated distinct clusters between the groups treated with antibiotics and *B. licheniformis*-fermented products. The bacterial richness and evenness in the feces decreased in weaning piglets fed 1 g/kg of *B. licheniformis*-fermented products and antibiotics. The abundance of the genera [*Ruminococcus*] *gauvreauii group*, *Ruminococcaceae UCG-005*, and *Ruminococcaceae UCG-008* in the feces decreased in weaning piglets fed *B. licheniformis*-fermented products or antibiotics. The average abundance of the genus *Prevotella 9* in the feces was positively correlated with the concentration of *B. licheniformis*-fermented products and negatively correlated with the diarrhea incidence in weaning piglets. Furthermore, the average abundance of the genus *Prevotella 9* in the feces was positively correlated with the growth performance of weaning piglets. These results demonstrate that *B. licheniformis*-fermented products can improve diarrhea incidence and fecal microflora composition in weaning piglets.

## 1. Introduction

The weaning period is an important time in the management of piglets. Postweaning diarrhea is the most frequent cause of heavy economic losses in pig herds [1]. The use of antibiotic growth promoters (AGP) has been effective in decreasing the incidence of diarrhea in weaning piglets [2]. However, the overuse of AGP in animal feed led to the evolution of drug-resistant bacteria and antibiotic residues in pigs. AGP have been banned in animal production in the European Union since 2006 and this policy is expected to expand to other countries. Hence, finding alternative solutions for preventing diarrhea in weaning piglets is urgent.

Intestinal microflora plays a critical role in the process of nutrient digestion and immunomodulation. Normal gut microbiota is responsible for resistance to colonization by exogenous pathogenic microorganisms [3]. Pathogen invasion alters the intestinal microbiota composition and causes diarrhea in weaning piglets [1]. In addition, stress or dysbiosis during the weaning period also results in an imbalanced gut microbiota, which also leads to postweaning diarrhea [4]. AGP could decrease postweaning diarrhea incidence by reducing pathogenic bacteria and modifying the gut microflora in piglets [5].

Probiotics have garnered interest as the alternative to AGP [6,7,8,9]. Probiotics can inhibit the growth of enteric pathogens and the development of subsequent diseases by producing antimicrobial substances in livestock [10,11,12,13]. A recent study reported that *Lactobacillus* species and *Clostridium butyricum*-fermented products alleviate diarrhea incidence and reduce the gut pathogens in weaning piglets [14]. Dietary *Bacillus licheniformis* and *Bacillus subtilis* complex supplementation increase nutrient digestibility and fecal *Lactobacillus* counts in growing-finishing pigs [15]. A *Bacillus* species mixture, including *B. licheniformis*, was able to modify the gut microbial diversity of piglets [16]. It has been demonstrated that *B. licheniformis* has antimicrobial activity against pathogens through the production of antibacterial cyclic lipopeptide [17,18]. Our previous study demonstrated that *B. licheniformis*-fermented products also exhibit antibacterial activity against *Clostridium perfringens* and *Staphylococcus aureus* in vitro [18]. Similar to AGP, *B. licheniformis*-fermented products promote growth performance, modulate gut microbiota, and mitigate *Clostridium perfringens*-induced necrotic enteritis in broilers [18,19]. However, to the best of our knowledge, no study has been conducted to examine the effects of *B. licheniformis*-fermented products on improvement in diarrhea incidence and gut microbiota in weaning piglets. Therefore, in the present study we set out to prove the hypothesis that *B. licheniformis*-fermented products can alleviate the diarrhea incidence and alter the gut microflora in weaning piglets. To this end, we investigated the effects of different levels of *B. licheniformis*-fermented products on the diarrhea incidence and fecal microflora composition of weaning piglets.

## 2. Materials and Methods

All experiments were performed in accordance with approved guidelines. The animal protocol was approved by the Institutional Animal Care and Use Committee of National Ilan University (IACUC, protocol number 108-6).

### 2.1. Preparation of B. licheniformis-Fermented Products

*B. licheniformis* was purchased from the Food Industry Research and Development Institute (ATCC 12713, Hsinchu, Taiwan). Wheat bran and soybean meal-based substrates were mixed with water in a space bag and the mixture was autoclaved at 121 °C and 15 psi of steam pressure for 15 min. The cooled solid-state fermentation substrates were inoculated with 4% (*v*/*w*) inoculum of *B. licheniformis* and incubated at 30 °C for 6 days in a chamber with free oxygen and relative humidity above 80%. After fermentation, fermented products were dried at 50 °C for 2 days to a moisture content of 10% and homogenized by mechanical agitation. The *B. licheniformis*-fermented products were then stored at 4 °C prior to analysis. Details of the preparation of *B. licheniformis*-fermented products are provided in a previous study [18]. The *B. licheniformis* quantities and antibacterial cyclic lipopeptide (surfactin) concentrations in fermented products were 3 × 10^12^ CFU/g and 4.7 mg/g, respectively [19].

### 2.2. Animal Study

One hundred and twenty crossbred ((Landrace × Yorkshire) × Duroc) 28-day-old, castrated male and female piglets with an average initial body weight of 9.87 ± 1.43 kg participated in the study. Piglets were randomly allotted one of 4 treatments (with 3 replicates of 10 piglets per stall) in a completely randomized design. The experimental diets consisted of (1) a basal diet with no treatment as control (C), (2) a basal diet plus 1 g/kg of *B. licheniformis*-fermented products (3 × 10^9^ CFU/kg of feed) (L), (3) a basal diet plus 4.5 g/kg of *B. licheniformis*-fermented products (1.35 × 10^10^ CFU/kg of feed) (H), and (4) a basal diet plus 30 mg/kg antibiotics (bacitracin methylene disalicylate) (A). Diets (Table 1) were formulated to meet or exceed the nutrient requirements recommended by the National Research Council (Nutrient Requirements for Swine, 2012). The experimental period was 28 d. All piglets were housed in stalls (2.5 × 4.0 m) with slatted plastic floors. The piglets were adapted to the basal diets and the housing for one week before starting the 28-day experiment. Room temperature was set at 30 °C at the beginning of the study and then gradually reduced to 24 °C by the end. The lighting schedule was 10 L: 14D throughout the experiment. Food and water were offered ad libitum throughout the experimental period. The individual body weight, average daily feed intake, average daily weight gain, and feed conversion ratio were recorded every week. The average final body weight of piglets was 27.34 ± 0.85 kg.

### 2.3. Diarrhea Incidence Analysis

Diarrhea incidence was recorded daily in each stall throughout the entire experiment through direct observation conducted by 2 evaluators. The diarrhea was determined based on fecal consistency using a modification of the method described by a previous study [20]. The feces were ranked on the following scale: 0 = solid; 1 = semi-solid; 2 = semi-liquid; and 3 = liquid. The diarrhea was defined as production of feces at level 2 or 3 for 2 continuous days. The piglets were considered to have diarrhea when the fecal consistency was level 2 or 3, as described previously by Cheng et al. [14] and calculated as follows:

Diarrhea incidence (%) = [the number of pigs with diarrhea in each stall × diarrhea d/(10 pigs × 28 d)] × 100.
(1)

### 2.4. 16S rRNA Sequencing and Data Processing

At the end of experiment, feces from 2 piglets per replicate were freshly collected by massaging the rectums and then pooled. Three replicates (6 piglets/treatment, *n* = 3) were used for fecal microbiota analysis. Total genomic DNA from feces was purified using a commercial DNA extraction kit (Zymo Research, Irvine, CA, USA). DNA concentration and purity were assessed through spectrophotometry and agarose gel electrophoresis. DNA amplicons from individual piglet samples were amplified with specific primers for the V3-V4 regions of the 16S rRNA gene through PCR. PCR products were purified using a commercial DNA purification kit (QIAGEN, Germantown, MD, USA) and sequencing libraries were constructed using TruSeq Nano DNA Library Prep Kits (Illumina, San Diego, CA, USA). The quality of the library was assessed on a Qubit 2.0 Fluorometer (Thermo Scientific, Waltham, MA, USA) and an Agilent Bioanalyzer 2100 system. The library was then sequenced on an Illumina MiSeq platform and an average read length of 300 bp was generated. The sequences were clustered into operational taxonomic units (OTUs) at 97% identity using the cluster program. The alpha and beta diversity was analyzed using QIIME 2 (Version 2017.4) software and the RDP classifier Bayesian Algorithm (http://rdp.cme.msu.edu/), respectively. The alpha diversity was analyzed by a species richness estimator (Chao1 and Fisher alpha) and a species evenness estimator (Shannon and Enspie). The beta diversity was analyzed using principal component analysis and principal coordinate analysis on UniFrac distance matrices. A Venn diagram (Version 1.6.17) was used to present the similarity and difference in OTUs between the 4 groups. Color correlograms were generated using the corrplot package in R (Version 0.84).

### 2.5. Statistical Analysis

Data were analyzed using one-way ANOVA through the GLM procedure in SAS software (Version 9.4; SAS Institute, Cary, NC, USA). Replicates were considered to be the experimental units. The results are expressed as mean ± SEM. Means were compared using Tukey’s HSD test at a significance level of *p* < 0.05. The relationship between abundant genera, concentration of *B. licheniformis*-fermented products, diarrhea incidence, and growth performance in weaning piglets of different groups was analyzed by Pearson’s correlation coefficient (r).

## 3. Results

### 3.1. Effect of B. licheniformis-Fermented Products on Diarrhea Incidence in Weaning Piglets

The effect of *B. licheniformis*-fermented products on the growth performance of weaning piglets is described in Table 2. The results revealed that *B. licheniformis*-fermented products did not affect body weight, daily weight gain, daily feed intake, and feed conversion ratio in weaning piglets compared with the control group. Growth performance was not improved by antibiotics supplementation in weaning piglets compared with the control group during the experimental period. However, the incidence of diarrhea in antibiotics-fed weaning piglets significantly decreased compared with the control group during the experimental period (*p* < 0.05) (Table 3). Similarly, 4.5 g/kg of *B. licheniformis*-fermented product-fed weaning piglets exhibited a lower incidence of diarrhea compared with the control group during the experimental period (*p* < 0.05) (Table 3). No significant difference was observed in the incidence of diarrhea between 4.5 g/kg of *B. licheniformis*-fermented product-fed and antibiotics-fed weaning piglets.

### 3.2. Effect of B. licheniformis-Fermented Products on the Fecal Bacterial Microbiota of Weaning Piglets

**Figure 1:**
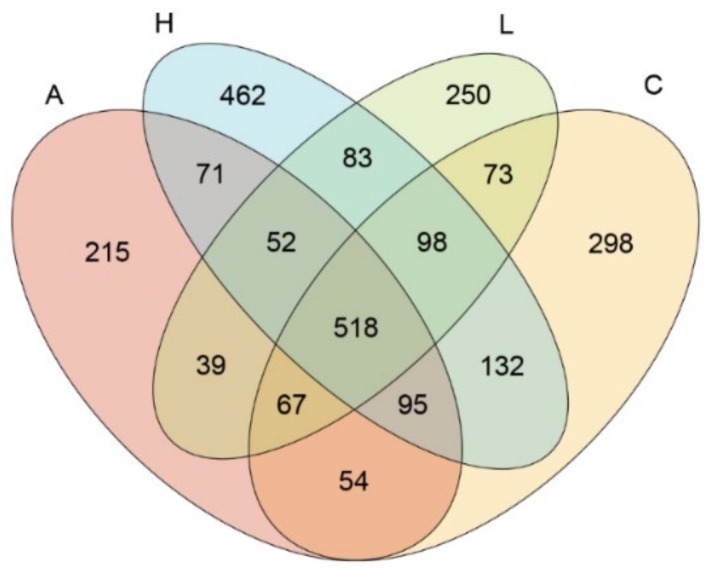
Operational taxonomic unit distribution and composition analysis of fecal content. A Venn diagram of the operational taxonomic unit (OTU) distribution of the fecal contents. Each ellipse represents one group. The overlapping regions between the ellipses represent the OTU that is shared between the following: basal diet as the control (C), basal diet plus 1 g/kg of *B. licheniformis*-fermented products (L), basal diet plus 4.5 g/kg of *B. licheniformis*-fermented products (H), and basal diet plus 30 mg/kg antibiotics (bacitracin methylene disalicylate) (A) (*n* = 3). The value of each region represents the number of OTUs corresponding to the region.

**Figure 2:**
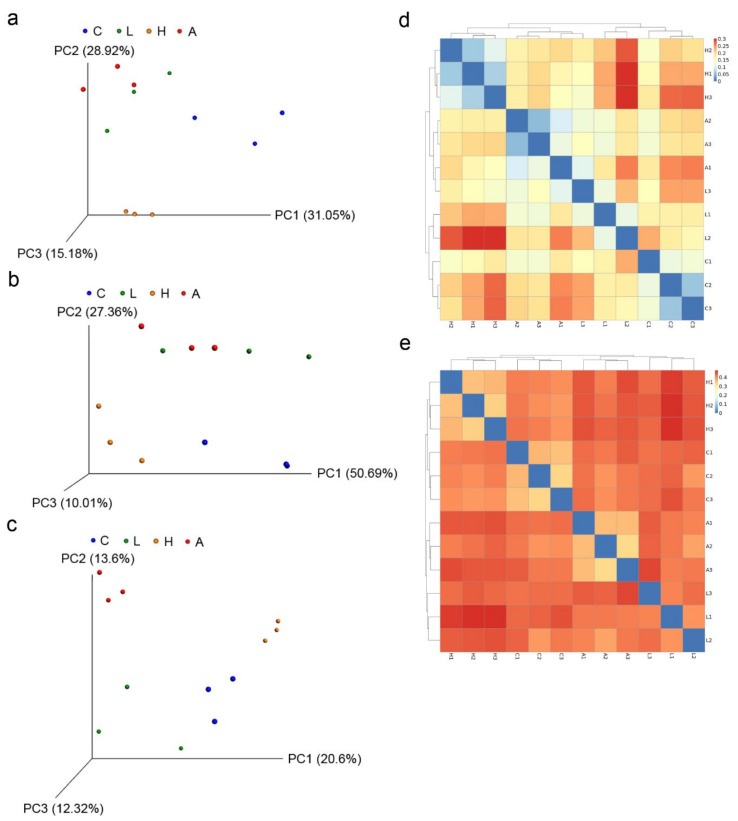
Comparison of the bacterial communities of the fecal contents by advanced analysis. (**a**) Principal component analysis plots of the fecal contents of basal diet as the control (C), basal diet plus 1 g/kg of *B. licheniformis*-fermented products (L), basal diet plus 4.5 g/kg of *B. licheniformis*-fermented products (H), and basal diet, 30 mg/kg antibiotics (bacitracin methylene disalicylate) (A) (*n* = 3). Principal coordinate analysis of (**b**) weighted UniFrac and (**c**) unweighted UniFrac distance of the fecal bacterial communities from C, L, H, and A (*n* = 3). The beta diversity index of the fecal contents from C, L, H, and A based on (**d**) weighted and (**e**) unweighted UniFrac metrics (*n* = 3).

The effect of *B. licheniformis*-fermented products on the fecal microbiota of weaning piglets is presented in Table 4. After stringent quality trimming of raw data, the averages of high-quality reads from the fecal content of weaning piglets fed only a basal diet, 1 g/kg of *B. licheniformis*-fermented products, 4.5 g/kg of *B. licheniformis-*fermented products, or antibiotics (hereafter referred to in sequence as the “4 aforementioned groups”) were 23,935, 23,598, 24,145, and 21,555, respectively. The average bacterial sequences from the fecal content in the 4 aforementioned groups were 4408, 3108, 5136, and 3085 OTUs, respectively. These results indicate decreased species richness, as estimated using the Chao 1 estimator, in the fecal content of the 1 g/kg of *B. licheniformis*-fermented products and antibiotics-treated group (*p* < 0.05). Similar results were obtained from Fisher alpha analysis; species richness was lower (relative to the control group) in the fecal content of the groups that were fed 1 g/kg of *B. licheniformis*-fermented products and antibiotics (*p* < 0.05). Furthermore, Shannon and Enspie analysis indicated that fecal species evenness was lower in the groups treated with 1 g/kg of *B. licheniformis*-fermented products and antibiotics relative to the control group (*p* < 0.05). The Venn diagram illustrated a greater overlap (518 OTUs, core) that was shared by 4 of the plotted groups (Figure 1). In total, 298, 462, 250, and 215 unique OTUs were discovered in the 4 aforementioned groups, respectively. Specifically, 73 OTUs were discovered in both the control group and group treated with 1 g/kg of *B. licheniformis*-fermented products; 132 OTUs were discovered in both the control group and group treated with 4.5 g/kg of *B. licheniformis*-fermented products. By contrast, 54 OTUs were discovered in both the control group and antibiotics-treated group. Principal component analysis conducted to examine the functional distinction of microbiota revealed statistically significant discrimination among the groups (PC1, 31.05%; PC2, 28.92%; PC3, 15.18%; Figure 2a). Principal coordinate analysis (PCoA) based on a weighted UniFrac metric indicated that the microbiota of fecal samples was clearly differentiated among the groups (PC1, 50.69%; PC2, 27.36%; PC3, 10.01%; Figure 2b). Similar results were also observed from PCoA based on an unweighted UniFrac metric (PC1, 20.6%; PC2, 13.6%; PC3, 12.32%; Figure 2c). Beta diversity analysis based on weighted and unweighted UniFrac metrics also indicated that the microbiota of fecal samples was clearly differentiated (Figure 2d,e).

### 3.3. Effects of B. licheniformis-Fermented Products on the Fecal Bacterial Taxonomic Composition

**Figure 3:**
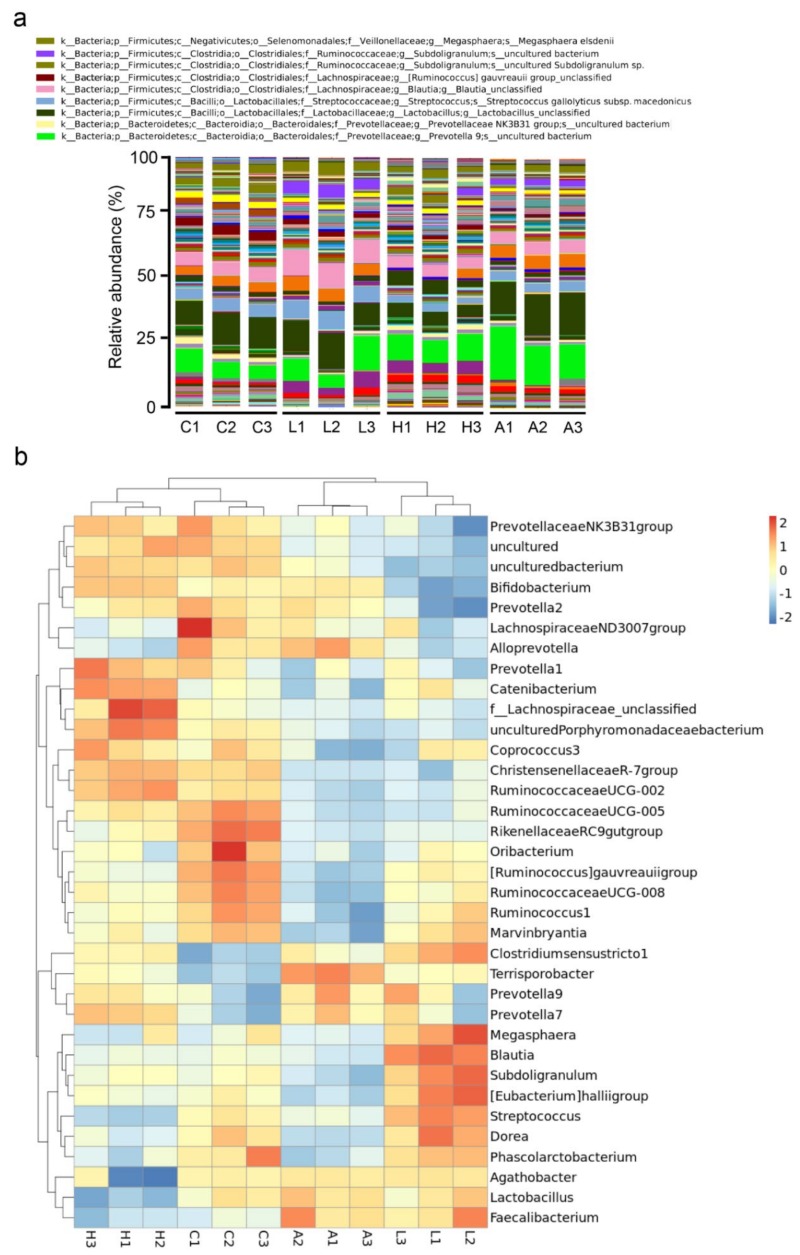
Bacterial taxonomic composition analysis of fecal content. (**a**) Genus-level composition of the microbiome from fecal content. Composition of major taxonomic groups at the genus level in samples collected from the basal diet as control (C), basal diet plus 1 g/kg of *B. licheniformis*-fermented products (L), basal diet plus 4.5 g/kg of *B. licheniformis*-fermented products (H), and basal diet plus 30 mg/kg antibiotics (bacitracin methylene disalicylate) (A) (*n* = 3). (**b**) Heatmap of species abundance of the microbiome from fecal content. Abundance distribution of dominant 35 genera (*Y*-axis) across all samples (*X*-axis) were displayed in the species abundance heatmap (*n* = 3). Values are normalized by Z-score.

The effect of *B. licheniformis*-fermented products on the bacterial taxonomy in the fecal contents of weaning piglets is described in Table 5. No significant differences were observed in the abundance of the phyla Firmicutes and Bacteroidetes among groups. Relative to the control group, at the phylum level, the abundance of the phylum Actinobecteria was higher in the group treated with 4.5 g/kg of *B. licheniformis*-fermented products (*p* < 0.05). In contrast, the abundance of the phylum Actinobecteria was decreased in the group treated with antibiotics (*p* < 0.05). The abundance of the phylum Proteobacteria was higher in the groups treated with 4.5 g/kg of *B. licheniformis*-fermented products relative to the control group (*p* < 0.05). Relative to the control group, at the class level, the proportions of the Clostridia class were reduced in the feces of weaning piglets fed basal diets that were supplemented with antibiotics (*p* < 0.05). The feces of weaning piglets fed 4.5 g/kg of *B. licheniformis*-fermented products had the lowest abundance of the Bacilli class and highest abundance of the Erysipelotrichia class (*p* < 0.05). Relative to the control group, the feces of weaning piglets fed antibiotics had the lowest abundance of the Methanobacteria class (*p* < 0.05). The feces of weaning piglets fed 1 g/kg of *B. licheniformis*-fermented products had the lowest abundance of the Actinobacteria class (*p* < 0.05). In contrast, the highest abundance of the Actinobacteria class was observed in the feces of weaning piglets fed 4.5 g/kg of *B. licheniformis*-fermented products (*p* < 0.05). At the order level, the proportions of the Clostridiales and Methanobacteriales order were lower in the feces of weaning piglets fed basal diets that were supplemented with antibiotics relative to those that only received basal diets (*p* < 0.05). The feces of the 4.5 g/kg of *B. licheniformis*-fermented product-treated group had the lowest and highest (both *p* < 0.05) abundance of the Lactobacillales and Erysipelotrichales orders, respectively. The feces of weaning piglets fed 1 g/kg of *B. licheniformis*-fermented products had the lowest abundance of the Bifidobacteriales order (*p* < 0.05), while 4.5 g/kg of *B. licheniformis*-fermented product-treated group had the highest abundance of the Bifidobacteriales order (*p* < 0.05). At the family level, the proportions of the Lachnospiraceae and Streptococcaceae families were higher in the 1 g/kg of *B. licheniformis*-fermented product-treated group (*p* < 0.05). The lowest abundance of the Ruminococcaceae and Lactobacillaceae families was observed in the feces of weaning piglets fed antibiotics and 4.5 g/kg of *B. licheniformis*-fermented products, respectively (*p* < 0.05). The feces of weaning piglets fed 1 g/kg of *B. licheniformis*-fermented products or antibiotics had the lowest abundance of the Muribaculaceae and Christensenellaceae families (*p* < 0.05). The abundance of the Clostridiaceae and Erysipelotrichaceae families in the feces of the 4.5 g/kg of *B. licheniformis*-fermented product-treated group was the highest (*p* < 0.05). Relative to the control group, the proportions of the Peptostreptococcaceae family in the antibiotics-treated group were increased (*p* < 0.05). At the genus level, the abundance of the *Lactobacillus*, *Streptococcus*, and *Agathobacter* genera was the lowest in the feces of weaning piglets fed 4.5 g/kg of *B. licheniformis*-fermented products (*p* < 0.05). The abundance of the *Blautia* and *Subdoligranulum* genera was the highest in the feces of weaning piglets fed 1 g/kg of *B. licheniformis*-fermented products (*p* < 0.05). The abundance of the *Prevotellaceae NK3B31 group* genus in the 1 g/kg of *B. licheniformis*-fermented product-treated group was the lowest (*p* < 0.05). The proportions of the genera *[Ruminococcus] gauvreauii group* and *Ruminococcaceae UCG-008* in the antibiotics-treated group were lower than that in the control group (*p* < 0.05). The abundance of the *Ruminococcaceae UCG-005* and *Prevotella 1* genera were the lowest in the feces of weaning piglets fed 1 g/kg of *B. licheniformis*-fermented products or antibiotics (*p* < 0.05). The abundance of the *Lachnospiraceae_unclassified* and *Prevotella 7* genera in the group that was treated with 4.5 g/kg of *B. licheniformis*-fermented products was higher than that in the control group (*p* < 0.05). An overview of the taxonomy at the genus level is also presented in Figure 3a. Based on the heatmap of the 35 most abundant genera, 2 distinct clusters were observed between the control and antibiotics-treated groups (Figure 3b). In addition, bacterial community clusters were partially shared between the 1 g/kg of *B. licheniformis*-fermented products and 4.5 g/kg of *B. licheniformis*-fermented products-treated group. Among the 35 most abundant genera, 8 genera (*LachnospiraceaeNO3007group*, *Ruminococcaceae UCG-005*, *RikenellaceaeRC9gutgroup*, *Oribacterium*, *[Ruminococcus] gauvreauii group*, *Ruminococcaceae UCG-008*, *Ruminococcus1*, and *Marvinbryantia*) were more abundant in the control group, and 6 genera (*Megasphaera*, *Blautia*, *Subdoligranulum*, *[Eubacerium]halliigroup*, *Streptococcus*, and *Dorea*) were enriched in only the groups treated with 1 g/kg of *B. licheniformis*-fermented products. Seven genera (*Prevotella1*, *Catenibacterium*, *f_Lachnospiraceae_unclassified*, *unculturedPorphyromonadaceaebacterium*, *Coprococcus3*, *ChristensenellaceaeR-7group*, and *Ruminococcaceae UCG-002*) were the most abundant in the group treated with 4.5 g/kg of *B. licheniformis*-fermented products.

### 3.4. Association between the Concentration of B. licheniformis-Fermented Products, Diarrhea Incidence, Growth Performance, and Average Abundance of the Genera

**Figure 4:**
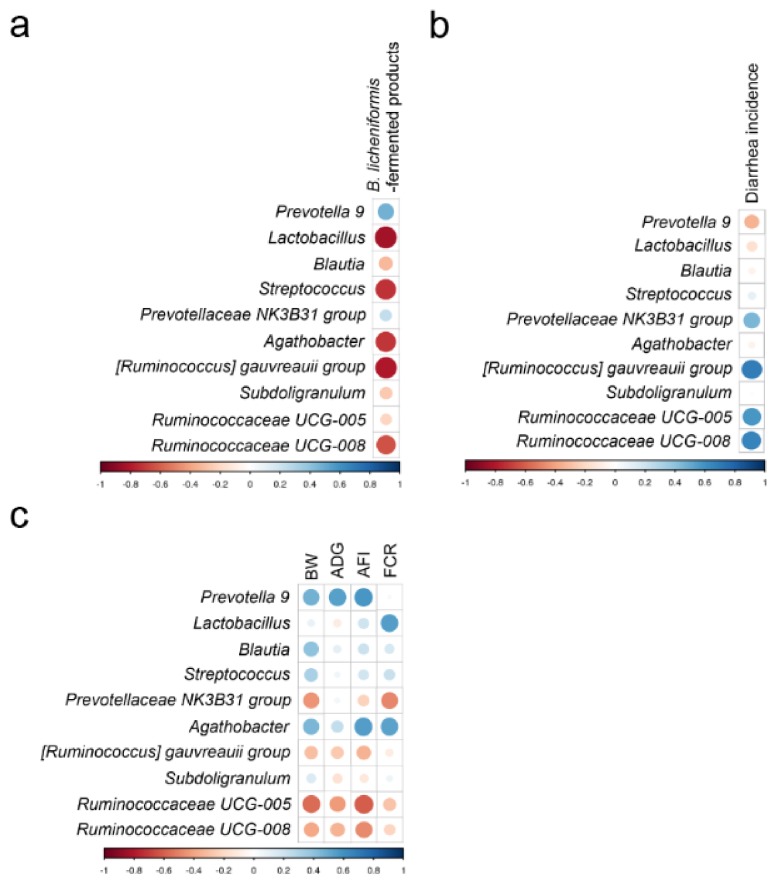
Correlation analysis of fecal microbiota. (**a**) Correlation analysis between concentration of *B. licheniformis*-fermented products and abundant genera in piglets of different groups. (**b**) Correlation analysis between diarrhea incidence and abundant genera in piglets of different groups. (**c**) Correlation analysis between growth performance and abundant genera in piglets of different groups. Circle sizes and color intensity represent the magnitude of correlation. Blue circle represents positive correlations; red circle represents negative correlations. BW = body weight, ADG = average daily gain, AFI = average daily feed intake, FCR = feed conversion ratio.

The results of correlation analysis between the concentration of *B. licheniformis*-fermented products, diarrhea incidence, growth performance and the abundant genera in the weaning piglets of different groups are presented in Figure 4. The average abundance of the genera *Prevotella 9* and *Prevotellaceae NK3B31 group* was positively correlated with the concentration of *B. licheniformis*-fermented products, whereas the other genera were negatively correlated with the concentration of *B. licheniformis*-fermented products (Figure 4a). The average abundance of the genera *Prevotella 9* and *Lactobacillus* was negatively correlated with diarrhea incidence, whereas the genera *Prevotellaceae NK3B31 group*, *[Ruminococcus] gauvreauii group*, *Ruminococcaceae UCG-005*, and *Ruminococcaceae UCG-008* were positively correlated with diarrhea incidence (Figure 4b). The genera *Prevotella 9* and *Agathobacter* were positively correlated with body weight (BW), average daily gain (ADG), and average feed intake (AFI), whereas the genera of *Ruminococcaceae UCG-005* and *Ruminococcaceae UCG-008* were negatively correlated with these 3 variables. In addition, the average abundance of the genera *Lactobacillus* and *Agathobacter* was positively correlated with feed conversion ratio (FCR), whereas the genera of *Prevotellaceae NK3B31 group*, *Ruminococcaceae UCG-005*, and *Ruminococcaceae UCG-008* were negatively correlated with FCR (Figure 4c).

## 4. Discussion

In this study, we demonstrated that 4.5 g/kg of *B. licheniformis*-fermented products reduced diarrhea incidence in weaning piglets. PCoA and the heatmap of species abundance indicated distinct clusters between the groups treated with antibiotics and *B. licheniformis*-fermented products. The abundance of the genera *[Ruminococcus] gauvreauii group*, *Ruminococcaceae UCG-005*, and *Ruminococcaceae UCG-008* was lower in the fecal content of the group treated with *B. licheniformis*-fermented products. The average abundance of the *Prevotella 9* genus was positively correlated with the concentration of *B. licheniformis*-fermented products and negatively correlated with diarrhea incidence in weaning piglets. Furthermore, the average abundance of the *Prevotella 9* genus was positively associated with the growth performance of weaning piglets.

A previous study demonstrated that a dietary combination of *Bacillus* species-based probiotics, containing *B. lichenformis*, *B. coagulans*, and *B. subtilis*, improves the growth performance in growing-finishing pigs [21]. It has been demonstrated that supplementation of *B. licheniformis* in combination with *B. subtilis* increases the growth performance and reduces the morbidity and mortality rates associated with diarrhea in weaning piglets [22]. Dietary *C. butyricum* in combination with *B. licheniformis* could reduce the diarrhea incidence in weaning piglets and partially replace the use of in-feed AGP and zinc oxide [23]. Much of the past research has focused on the effects of multi-strains of *Bacillus* species on the growth performance and diarrhea incidence in weaning piglets. In our study, we demonstrated that, similar to the effects of antibiotics, the dietary supplementation of 4.5 g/kg of *B. licheniformis*-fermented products could alleviate diarrhea incidence in weaning piglets. In addition, the *B. licheniformis* formula used in the present study is different from other studies. Here, we treated piglets with *B. licheniformis*-fermented products. *B. licheniformis*-fermented products not only contain live microorganisms but also have *B. licheniformis*-derived antibacterial cyclic lipopeptide, the surfactin. Therefore, we hypothesize that the efficiency of *B. licheniformis*-fermented products on improvement of diarrhea incidence of weaning piglets may be different when compared with other studies. Taken together, the results indicate that the dietary supplementation of *B. licheniformis*-fermented products has beneficial effects on the prevention of postweaning diarrhea.

The gut microbiota plays an important role in utilizing nutrients, producing volatile fatty acids and vitamins, modulating the immune system and enhancing resistance against enteric pathogens. Gut microbial diversity and composition can be shaped by feed additives, such as antibiotics and probiotics [24]. It has been demonstrated that antibiotic supplementation leads to an imbalanced microbiome in the gastrointestinal tract of humans and poultry [19,25]. In our study, we demonstrated that supplementation of antibiotics in the diet also had a negative effect on the richness and diversity of the fecal microbiota of piglets. It has been reported that the richness and evenness of the microbiota were decreased in the feces of pigs fed *Bacillus* species-based probiotics [16,26]. Similarly, 1 g/kg of *B. licheniformis*-fermented products also reduce the richness and evenness of the fecal microbiota in piglets in the present study. Our recent study has demonstrated that the bacterial richness and evenness was reduced in the fecal content of broilers in response to *B. licheniformis*-fermented products in a dose-dependent manner [19]. Interestingly, no obvious negative effects on bacterial richness and evenness were observed in the fecal content of the group that was fed 4.5 g/kg of *B. licheniformis*-fermented products. The abundance of *Lactobacillus* species and *Bifidobacterium* species were increased in the feces of growing pigs fed *Bacillus* species-based probiotics [26]. A linear trend of *Bacillus* species-based probiotics supplementation on the number of *Lactobacillus* species was found [21]. Dietary supplementation of *B. licheniformis* in combination with *B. subtilis* reprograms the microbiota of weaning piglets challenged with F4 positive enterotoxigenic *E. coli.* strain (F4^+^ ETEC) [27]. Low-doses of *Bacillus* species-based probiotics also increase the number of *Lactobacillus* species in the feces of weaning piglets challenged with F4^+^ ETEC, while high-doses of *Bacillus* species-based probiotics have a negative impact on the number of *Lactobacillus* species [27]. Here, we also found that 4.5 g/kg of *B. licheniformis*-fermented products could reduce the number of *Lactobacillus* species in the feces of weaning piglets compared to the feces of piglets fed 1 g/kg of *B. licheniformis*-fermented products. In addition to the number of *Lactobacillus* species, the abundance of phylum Proteobacteria was increased in response to high-doses of *Bacillus* species-based probiotics treatment in the feces of weaning piglets challenged with F4^+^ ETEC [27]. Similar results were also observed in the fecal content of the group that was fed 4.5 g/kg of *B. licheniformis*-fermented products in the current study. These findings suggest that a distinct bacterial community cluster was formed between the groups treated with antibiotics and *B. licheniformis*-fermented products. The dosage of *B. licheniformis*-fermented products exhibited differential effects on the fecal microbiota community in weaning piglets.

A previous study reported that at the genus level, the proportion of the *Lactobacillus* and *Prevotella* genus was increased in weaning piglets compared to suckling piglets [24]. *Lactobacillus* species could improve nutrient utilization, gut health, growth performance, and the immune system in piglets [28]. Our previous study also demonstrated that the abundance of the *Lactobacillus* genus in the feces was increased in broilers with a low feed conversion ratio in the groups treated with *B. licheniformis*-fermented products [19]. Here, we found that the abundance of the *Lactobacillus* genus in the feces was increased in response to 1 g/kg *B. licheniformis*-fermented products. However, the abundance of the *Lactobacillus* genus in the feces of piglets was not linearly correlated with the concentration of *B. licheniformis*-fermented products. It has been reported that excessive supplementation of *B. licheniformis* in combination with *B. subtilis* may disrupt the gut microbial ecology, thereby decreasing the abundance of the *Lactobacillus* genus in the feces of piglets [27]. *Prevotella* species are able to degrade polysaccharides in the plant cell wall by producing digestive enzymes and then produce short-chain fatty acids in the gut of growing pigs [29,30]. In addition, *Prevotella* species may improve the feed intake and nutrient utilization in the piglets [31,32]. The abundance of the *Prevotella* genus in the feces of pigs had a negative relationship to feed conversion ratio [33]. In our study, we demonstrated that the abundance of the genus *Prevotella* 9 in the feces of weaning piglets was negatively correlated with diarrhea incidence and positively correlated with the growth performance (BW, ADG, and AFI). Furthermore, supplementation of *B. licheniformis* could increase the abundance of the genus *Prevotella* 9 in the feces of weaning piglets. It has been reported that the abundance of the genus *Ruminococcus gauvreauii* was negatively associated with weaning body weight in meat rabbits [34]. *Ruminococcus gauvreauii* and *Ruminococcaceae UCG-005* are positively correlated with chronic inflammation, metabolic diseases, and mycotoxin exposure in rats and weaning piglets [35,36]. In the present study, we found that the abundance of the *[Ruminococcus] gauvreauii group*, *Ruminococcaceae UCG-005*, and *Ruminococcaceae UCG-008* were positively correlated with diarrhea incidence and negatively correlated with growth performance (BW, ADG, and AFI). These results imply that the abundance of the *Prevotella* 9 and *Ruminococcus* genera in the gastrointestinal tract is a significant factor in the growth performance and diarrhea incidence in weaning piglets.

## 5. Conclusions

4.5 g/kg of *B. licheniformis*-fermented products potentially improve diarrhea incidence and modulates the fecal microbiota of weaning piglets. A distinct bacterial community cluster was found between the group treated with *B. licheniformis*-fermented products and the antibiotics-treated group. These findings are particularly important for a better understanding of how *B. licheniformis*-fermented products and antibiotics differentially affect the fecal microbiota of weaning piglets and might be used as an alternative to antibiotics in the future for microbiota manipulation in order to improve postweaning diarrhea.

## Figures and Tables

**Table 1:** Composition of basal diets.

Item	d 1–28 ^4^
Ingredient, g kg^−1^	
Corn, yellow	570.0
Soybean meal, 44% CP	260.0
Fish meal, 60% CP	30.0
Fermented soybean meal	0
Dried whey	85.0
Soybean oil	35.0
CaCO_3_, 38% Ca	5.0
CaHPO_4_	12.0
Salt	4.0
Choline, 50%	0.8
L-Lysine, 98%	3.5
Vitamin, premix ^1^	1.0
Mineral, premix ^2^	1.0
Chemical composition, g kg^−1^	
Crude protein	191.2
Analyzed calcium	8.0
Analyzed total phosphorus	7.0
Lysine	14.5
Methionine + Cystine	6.5
Phenylalanine + Tyrosine	15.5
ME, kcal/kg ^3^	3567.63

^1^ Supplied per kg diet: vitamin A, 6000 IU; vitamin D, 900 IU; vitamin E, 30 IU; vitamin K3, 3 mg; vitamin B2, 6 mg; pantothenic acid, 18 mg; niacin, 60 mg; and vitamin B12, 30 μg, ^2^ Supplied per kg diet: Cu, 20 mg; Zn, 100 mg; Fe, 140 mg; Mn, 4 mg; Se, 0.1 mg; and I, 0.2 mg, ^3^ The metabolizable energy (ME) density of the diet from nutrient content was calculated based on the equation: ME = (35 × crude protein (%) + 85 × crude fat (%) + 35 × nitrogen-free extract (%)), ^4^ Fed in meal form to all pigs.

**Table 2:** Effect of *Bacillus licheniformis*-fermented products on growth performance of weaning piglets.

	C ^1^	L ^2^	H ^3^	A ^4^	SEM	*p*-Value
Body weight (kg/head)						
28 d	26.77	27.42	27.32	27.84	0.81	0.55
Average daily gain (g/d/head)						
1–14 d	0.53	0.53	0.54	0.55	0.04	0.80
15–28 d	0.70	0.72	0.69	0.71	0.03	0.87
1–28 d	0.61	0.63	0.62	0.63	0.03	0.85
Average daily feed intake (g/d/head)						
1–14 d	0.72	0.72	0.75	0.74	0.03	0.58
15–28 d	1.05	1.11	1.02	1.12	0.07	0.36
1–28 d	0.88	0.92	0.88	0.93	0.04	0.33
Feed conversion ratio						
1–14 d	1.38	1.36	1.38	1.35	0.04	0.69
15–28 d	1.50	1.54	1.49	1.58	0.06	0.42
1–28 d	1.45	1.47	1.44	1.48	0.03	0.61

Values are expressed as mean (*n* = 3). ^1^ C = Basal diet, ^2^ L = Basal diet plus 1 g/kg *Bacillus licheniformis*-fermented products, ^3^ H = Basal diet plus 4.5 g/kg *Bacillus licheniformis*-fermented products, ^4^ A = Basal diet plus 30 mg/kg antibiotics (bacitracin methylene disalicylate).

**Table 3:** Effect of *Bacillus licheniformis*-fermented products on diarrhea incidence among weaning piglets.

	C ^1^	L ^2^	H ^3^	A ^4^	SEM	*p*-Value
Diarrhea, %						
1–14 d	7.62	3.81	4.29	0.95	6.55	0.59
15–28 d	15.98	5.24	2.86	2.43	6.80	0.08
1–28 d	11.80 ^a^	4.52 ^a,b^	3.57 ^b^	1.69 ^b^	3.65	0.02

Values are expressed as mean (*n* = 3), ^a,b^ Means within a row that have no common superscript are significantly different (*p* < 0.05). ^1^ C = Basal diet, ^2^ L = Basal diet plus 1 g/kg *Bacillus licheniformis*-fermented products, ^3^ H = Basal diet plus 4.5 g/kg *Bacillus licheniformis*-fermented products, ^4^ A = Basal diet plus 30 mg/kg antibiotics (bacitracin methylene disalicylate).

**Table 4:** Sample information, microbial diversity, and sequence abundance in the fecal contents of weaning piglets.

	Effective Reads	Number of OTUs	Chao1	Fisher Alpha	Shannon	Enspie
C ^1^	23,934.67	4408.33 ^a,b^	816.00 ^a,b^	151.72 ^a,b^	6.89 ^a^	37.62 ^b^
L ^2^	23,597.67	3108.33 ^a,b^	660.67 ^b^	117.16 ^b^	6.10 ^b^	25.07 ^c^
H ^3^	24,145.33	5135.67 ^a^	915.00 ^a^	174.91 ^a^	7.22 ^a^	54.19 ^a^
A ^4^	21,554.67	3085.00 ^b^	671.67 ^b^	121.95 ^b^	6.20 ^b^	20.47 ^c^
SEM	1675.91	705.07	66.29	14.47	0.14	3.08
*p*-value	0.33	0.03	0.02	0.01	*<*0.001	*<*0.001

Values are expressed as mean (*n* = 3), ^a–c^ Means of a column with no common superscript are significantly different (*p* < 0.05). ^1^ C = Basal diet, ^2^ L = Basal diet plus 1 g/kg *Bacillus licheniformis*-fermented products, ^3^ H = Basal diet plus 4.5 g/kg *Bacillus licheniformis*-fermented products, ^4^ A = Basal diet plus 30 mg/kg antibiotics (bacitracin methylene disalicylate).

**Table 5:** Bacterial taxonomy within the fecal contents of weaning piglets.

	Relative Abundance (%)					
	C ^1^	L ^2^	H ^3^	A ^4^	SEM	*p*-Value
Phylum						
*Firmicutes*	67.15	72.20	63.51	75.87	5.34	0.11
*Bacteroidetes*	30.66	24.29	31.77	22.26	5.53	0.18
*Actinobacteria*	1.22 ^b^	1.14 ^b^	1.95 ^a^	0.78 ^c^	0.10	<0.001
*Proteobacteria*	0.32 ^b^	0.35 ^b^	0.64 ^a^	0.27 ^b^	0.07	<0.001
Class						
*Clostridia*	47.75 ^a^	47.83 ^a^	44.88 ^a,b^	40.37 ^b^	2.47	0.03
*Bacteroidia*	24.29	22.26	31.77	30.66	5.53	0.18
*Bacilli*	17.02 ^a^	19.62 ^a^	8.55 ^b^	20.15 ^a^	2.23	<0.01
*Negativicutes*	6.35	7.23	6.24	5.91	1.14	0.62
*Erysipelotrichia*	1.07 ^b^	1.18 ^b^	3.85 ^a^	0.72 ^b^	0.27	<0.001
*Methanobacteria*	1.32 ^a^	0.35 ^a,b^	1.21 ^a,b^	0.14 ^b^	0.35	0.02
*Actinobacteria*	0.66 ^c^	0.14 ^d^	1.35 ^a^	0.92 ^b^	0.05	<0.001
Order						
*Clostridiales*	47.75 ^a^	47.83 ^a^	44.88 ^a,b^	40.37 ^b^	2.47	0.03
*Bacteroidales*	24.26	22.22	31.73	30.65	5.53	0.18
*Lactobacillales*	17.02 ^a^	19.62 ^a^	8.55 ^b^	20.15 ^a^	2.23	<0.01
*Selenomonadales*	6.35	7.23	6.24	5.91	1.14	0.62
*Erysipelotrichales*	1.07 ^b^	1.18 ^b^	3.85 ^a^	0.72 ^b^	0.27	<0.001
*Methanobacteriales*	1.32 ^a^	0.35 ^a,b^	1.21 ^a,b^	0.14 ^b^	0.35	0.02
*Bifidobacteriales*	0.66 ^c^	0.14 ^d^	1.35 ^a^	0.92 ^b^	0.05	<0.001
Family						
*Prevotellaceae*	20.00	20.50	27.66	28.60	5.67	0.20
*Lachnospiraceae*	27.28 ^a,b^	30.18 ^a^	24.55 ^b^	24.49 ^b^	1.15	<0.01
*Ruminococcaceae*	16.55 ^a^	13.68 ^a,b^	14.81 ^a^	10.93 ^b^	1.56	0.01
*Lactobacillaceae*	12.23 ^a^	12.87 ^a^	5.71 ^b^	16.13 ^a^	2.02	<0.01
*Streptococcaceae*	4.79 ^b^	6.75 ^a^	2.84 ^c^	4.01 ^b^	0.32	<0.001
*Veillonellaceae*	5.40	6.27	5.66	5.33	1.02	0.71
*Muribaculaceae*	2.29 ^a^	1.02 ^b^	2.68 ^a^	1.35 ^b^	0.20	<0.001
*Rikenellaceae*	1.56 ^a^	0.52 ^b^	0.71 ^b^	0.50 ^b^	0.10	<0.001
*Christensenellaceae*	1.28 ^a^	0.39 ^b^	1.55 ^a^	0.41 ^b^	0.11	<0.001
*Clostridiaceae*	1.23 ^c^	1.77 ^a,b^	2.07 ^a^	1.66 ^b^	0.11	<0.001
*Erysipelotrichaceae*	1.07 ^b^	1.18 ^b^	3.85 ^a^	0.72 ^b^	0.27	<0.001
*Peptostreptococcaceae*	0.90 ^c^	1.40 ^b^	1.26 ^b,c^	2.45 ^a^	0.11	<0.001
Genus						
*Prevotella 9*	9.22	14.05	15.00	18.37	3.58	0.08
*Lactobacillus*	12.23 ^a^	12.87 ^a^	5.71 ^b^	16.13 ^a^	2.02	<0.01
*Blautia*	5.41 ^b^	10.17 ^a^	5.27 ^b^	5.15 ^b^	0.33	<0.001
*Streptococcus*	4.79 ^b^	6.75 ^a^	2.84 ^c^	4.01 ^b^	0.32	<0.001
*Prevotellaceae NK3B31 group*	3.46 ^a^	1.37 ^b^	3.39 ^a^	2.07 ^a,b^	0.57	<0.01
*Agathobacter*	4.08 ^a,b^	5.46 ^a^	1.48 ^b^	5.59 ^a^	1.03	0.02
*[Ruminococcus] gauvreauii group*	3.71 ^a^	2.10 ^b^	1.65 ^b,c^	1.23 ^c^	0.19	<0.001
*Subdoligranulum*	3.17 ^a^	4.72 ^b^	3.17 ^a^	2.58 ^a^	0.36 ^a^	<0.01
*Ruminococcaceae UCG-005*	2.94 ^a^	1.06 ^c^	1.98 ^b^	1.05 ^c^	0.19	<0.001
*Ruminococcaceae UCG-008*	2.74 ^a^	1.82 ^b^	1.82 ^b^	1.30 ^c^	0.15	<0.001
*Megasphaera*	3.08	4.69	2.88	2.85	0.65	0.05
*Lachnospiraceae unclassified*	1.95 ^b^	1.56 ^b^	4.67 ^a^	1.59 ^b^	0.85	0.02
*Prevotella 1*	1.45 ^a,b^	0.86 ^b^	2.37 ^a^	0.89 ^b^	0.45	0.03
*Prevotella 7*	1.30 ^b^	2.12 ^a,b^	3.65 ^a^	3.25 ^a,b^	0.67	0.02
*Lachnospiraceae ND3007 group*	1.25	0.82	0.81	1.00	0.18	0.15

Values are expressed as mean (*n* = 3), ^a–c^ Means within a row that have no common superscript are significantly different (*p* < 0.05). ^1^ C = Basal diet, ^2^ L = Basal diet plus 1 g/kg *Bacillus licheniformis*-fermented products, ^3^ H = Basal diet plus 4.5 g/kg *Bacillus licheniformis*-fermented products, ^4^ A = Basal diet plus 30 mg/kg antibiotics (bacitracin methylene disalicylate), ^5^.
